# Single Stage Transanal Pull-Through for Hirschsprung’s Disease in Neonates: Our Early Experience

**Published:** 2013-10-01

**Authors:** Pradeep Bhatiav, S Rakesh Joshi, Jaishri Ramji, Mitesh Bachani, Amit Uttarwar

**Affiliations:** Department of Pediatric Surgery, BJ Medical College, Ahmedabad, India

**Keywords:** Hirschsprung’s disease, Transanal pull through, Single stage surgery

## Abstract

**Objective:**

Hirschsprung’s disease is one of the common causes of intestinal obstruction in neonates. Transanal endorectal pull-through represents the latest development in the concept of the minimally invasive surgery for Hirschsprung’s disease. In this study, we present our early experience with single stage transanal pull through in neonates.

Design: Retrospective study of neonates with single stage transanal pull-through done for Hirschsprung’s disease in our institute from January 2011 to January 2013.

**Material and Method:**

Five newborn boys who presented with Hirschsprung’s disease were studied. The selection criteria included radiological transition zone at rectosigmoid or mid-sigmoid region, weight more than 2 kg, no evidence of enterocolitis or sepsis and no associated major anomaly. Single stage transanal endorectal pull-through was done in these patients. The follow-up period ranged from 6 months to 2 years.

**Results:**

Five patients with a mean age of 26.4 days (range 15-45 days) and a mean weight of 2.6 Kg (range 2.2 to 3.7 Kg) underwent transanal endorectal pull through. The mean operating time was 68 min (range 60 to 120 min). The average intra-operative blood loss was 20 ml (range – 10 to 30 ml) and the average length of bowel resected was 12.8 cm (range – 10 to 18 cm). Post-operatively patients passed first stool between 2nd and 3rd day. Oral feeding was resumed on 5th to 6th post-operative day. The average post-operative duration of stay in hospital was 10 days. None of the patients had post-operative bleeding, urethral injury, anastomotic leak or retraction of anastomotic site. Three patients developed perianal excoriation and one patient had post-operative enterocolitis. No mortality occurred in the series.

**Conclusion:**

Advancement in pediatric anaesthesia, availability of pediatric surgical expertise, improvement in pre-operative and post-operative management and nursing care has made single stage transanal pull-through in neonates a feasible option. The early results are comparable to single stage or multistage surgery in older children.

## INTRODUCTION

Hirschsprung’s disease is one of the common causes of intestinal obstruction in neonates. [1] There have been considerable advances in the management and correction of Hirschsprung’s disease since Swenson first described the pathological basis of the disease. [2] In the past, many of these children presented late with malnutrition, sepsis and colonic distension, but now a days most of them present within the neonatal age group.

Standard surgical teaching advocates that a proximal diverting colostomy should be done at the time of diagnosis and the child be allowed to grow before performing a definitive pull-through. It was also believed that operating on an older child made the pull-through technically easier. In modern practice, the early diagnosis of Hirschsprung’s disease makes it possible for patients to undergo definitive surgery at a younger age before they are debilitated by recurrent attacks of enterocolitis. Single stage transanal pull-through for the management of Hirschsprung’s disease was almost concurrently described by de la Torre-Mondregon, Ortega-Salgado [3] and by Langer et al [4] in 1998 and 1999 respectively. In neonates, this procedure is particularly beneficial as the dissection is easier due to the fact that the colon above the aganglionic segment is not much dilated and there are fewer or no episodes of enterocolitis. [5]

Though we have been doing transanal pull-through in older children for the last few years, we have recently started this procedure in neonates. In this study, we present our early experience with single stage transanal pull through in neonates and its feasibility with respect to peri-operative, post-operative course and early outcome.

## MATERIALS AND METHODS

Five newborn boys with radiologically confirmed recto-sigmoid Hirschsprung’s disease from January 2011 to January 2013 were retrospectively studied. All presented with neonatal intestinal obstruction. The initial management included nasogastric aspiration, intravenous fluids and rectal washouts, followed by single stage surgery. The inclusion criteria were clear evidence of radiological transition zone at the recto-sigmoid or mid-sigmoid region, weight more than 2 kg, no evidence of enterocolitis or sepsis and no associated major anomaly. The neonates with severe enterocolitis, bowel obstruction not responding to bowel decompression, preoperatively known long segment involvement or with major associated anomalies were excluded from study. Pre-operative bowel preparation was done using warm saline until effective decompression of the bowel was achieved. Single stage transanal pull-through was performed in all patients.

**Surgical technique:**

After induction of general anaesthesia, the patients were given injection ceftazidime intravenously. Foleys urethral catheter and nasogastric tube were inserted. The patient was placed in lithotomy position and everting sutures were taken around anus to expose the anal mucosa. The anal mucosa was incised circumferentially using needle tip cautery approximately 1 cm from the dentate line. Dissection was started in the submucosal plane for about 1-2 cm and then converted to full thickness of rectal wall beginning posteriorly and then continued circumferentially. The rectum was mobilized by working on the surface of the rectal wall using cautery. The dissection could be performed easily once the peritoneal reflection was reached and the rectum and sigmoid colon were mobilized out of anus leaving a muscular cuff of 1-2 cm. The dissection was continued till the transition zone and the proximal dilated colon was clearly identified. The aganglionic colonic segment was resected 3-5 cm proximal to the transition zone and a full thickness colo-anal anastomosis was performed with vicryl 5-0. Nearly 12-16 stitches were taken to complete the colo-anal anastomosis. Antibiotic soaked paraffin gauge was kept as anal pack and everting sutures were removed. The resected segment was sent for histopathological confirmation of aganglionosis.

The patients were kept nil by mouth for 5-6 days depending on bowel recovery and the feeding was slowly advanced to normal. The first rectal examination was performed under anaesthesia three weeks after the operation and the anastomotic site was assessed for any stricture, stenosis, or pus discharge. There after patients were followed up every 15 days for 3 months and assessed for the pattern of stooling, enterocolitis and weight gain. After 3 months, the follow up was continued at monthly intervals. The total follow-up period ranged from 6 months to 2 years.

## RESULTS

Five patients with a mean age of 26.4 days (range 15-45 days) and a mean weight of 2.6 Kg (range 2.2 to 3.7 Kg) underwent transanal endorectal pull through. The level of aganglionosis in all the cases was recto-sigmoid. The mean operating time was 68 min (range 60 to 120 min). The average intra-operative blood loss was 20 ml (range – 10 to 30 ml). The average length of bowel resected was 12.8 cm (range – 10 to 18 cm). [Table-I] In all the patients, the final histopathology report corresponded to the clinic-radiological findings.

**Figure:**
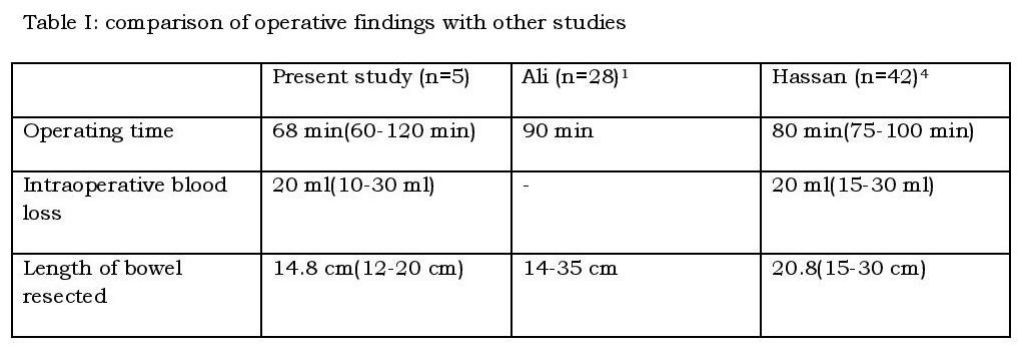
Table 1: Comparison of operative findings with other studies

Post-operatively, the first passage of stool was on 2nd to 3rd day; oral feeds were started on 5th to 6th post-operative day. The average post-operative duration of stay in hospital was 10 days. No mortality occurred in the series. [Table- II]

**Figure:**
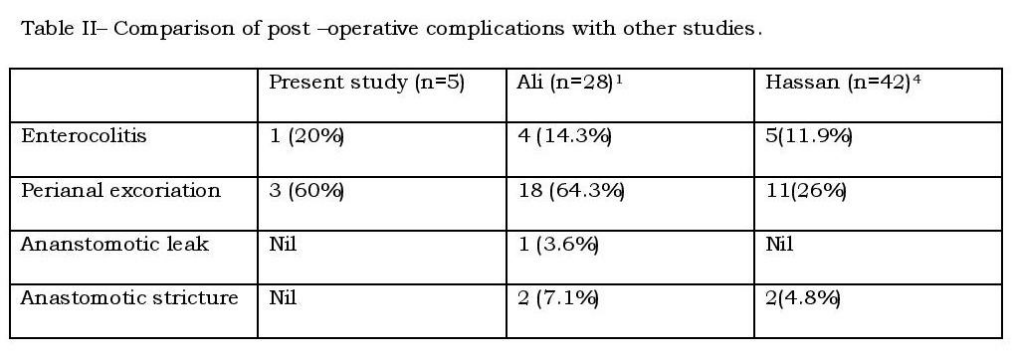
Table 2: Comparison of Postoperative complications with other studies

None of the patients had post-operative bleeding, urethral injury, anastomotic leak or retraction of anastomotic site. Three patients developed perianal excoriation and were managed with frequent application of zinc oxide cream. One patient had attack of enterocolitis in the 5th month after surgery.

## DISCUSSION

Hirschsprung’s disease is a common surgical problem in children. In majority of the children, the diseased segment extends up to the recto-sigmoid junction. Previously, multistage surgery was the preferred mode of management. Over the past two decades, it has been increasingly recognized that the routine use of a colostomy is unnecessary and an increasing number of pediatric surgeons perform the reconstruction as a single stage procedure at an early age.

Transanal endorectal pull-through represents the latest development in the concept of the minimally invasive surgery for Hirschsprung’s disease. The single stage management of Hirschsprung’s disease avoids colostomy and its related complications including the social stigma of a stoma. The short operating time and hospital stay with encouraging early results at affordable cost makes it the obvious choice over traditional multistage procedures. 

Transanal pull-through is relatively simple and advantageous in newborns, in whom the colon is loosely fixed to the retroperitoneum allowing longer segments of the colon to be resected through the anus in contrast to the more laborious dissection in older patients. [6] In our study the average length of bowel resected was 14.8 cm, which is comparable to other studies done [1, 5] [Table I]. The operating time and intraoperative blood loss are also lower in neonates as compared to older children due to the presence of less adherent mucosa, lesser fat laden mesentery, less dilated colon above the aganglionic segment, easily controllable blood vessels and less episodes of enterocolitis. [5] 

In the initial description of transanal endorectal pull through procedure, a long seromuscular cuff that reached the peritoneal reflection was left. This long cuff is often blamed for post-operative obstructive symptoms, constipation and enterocolitis. To avoid this problem, a few authors have used a shorter mucosectomy with a shorter muscular cuff measuring 1-2 cm above the dentate line. [7] A similar technique was used in this study. 

One of the major issues in the transanal approach is the significant stretching of the anal sphincters during surgery with its potential impact on continence in later life. However, this is only transient and bowel movements became normal in majority of cases within a period of 2 weeks to 3 months.[8] Van Leeuwen et al have reported that anorectal manometric studies were similar in both patients undergoing transanal endorectal pull-through and conventional procedures. [9]

Enterocolitis has been considered as one of the main problems in patients with Hirschsprung’s disease both before and after definite treatment. The incidence of post pull-through enterocolitis reported in the literature varies widely with some studies reporting rates as high as 32 to 42%. Hackman et al10 studied the risk factors for post-operative enterocolitis and found that both the presence of anastomotic leak or stricture and the development of post-operative intestinal obstruction secondary to adhesions increased the relative risk and subsequent enterocolitis by approximately 3 fold. Long seromuscular cuff and a high colo-anal anastomosis are associated with increased risk of post-operative enterocolitis. In this study, one patient developed enterocolitis in the follow-up period and was managed with intravenous fluids, rectal wash and intravenous antibiotics.

## CONCLUSION

Advancement in pediatric anaesthesia, availability of expert pediatric surgical facility, improvement in peri-operative and post-operative management and nursing care has made single stage transanal pull-through in neonates a feasible option. In neonates, the single stage surgery is safe and effective provided we follow proper selection criteria in form of weight of child, mode of presentation, associated anomalies and length of colonic segment involved. Early results are comparable to single stage and multistage surgery in older children. The additional advantages over the staged procedure are avoidance of additional hospital admissions, a shorter total hospital stay, a single anaesthetic operative procedure, immediate colonic continuity and satisfactory stool pattern with far superior cosmetic appearance. 

## Footnotes

**Source of Support:** Nil

**Conflict of Interest:** None

